# Assessment of Knowledge, Attitudes, and Practices Regarding Cervical Cancer and Its Vaccine Among Adolescent Girls at an Urban Health Training Center in Chhallapura, Datia, Madhya Pradesh: A Community-Based Study

**DOI:** 10.7759/cureus.111412

**Published:** 2026-06-24

**Authors:** Kalpana Arya, Rajju Tiwari, Vidhi Singh, Pradeep Sukla, Sanjeev Kumar, Shubhanshu Gupta, Ramlakhan Meena

**Affiliations:** 1 Community Medicine, Government Medical College, Datia, Datia, IND; 2 Biochemistry, Government Medical College, Datia, Datia, IND; 3 Obstetrics and Gynaecology, Government medical college, Datia, Datia, IND

**Keywords:** adolescent girls, cervical cancer, hpv, hpv vaccine, knowledge

## Abstract

Background

Cervical cancer remains a major public health problem, particularly in low- and middle-income countries (LMICs), with limited information on knowledge, attitudes, and practices (KAP) related to cervical cancer and human papillomavirus (HPV) vaccination among urban adolescents.

Objectives

To assess KAP regarding cervical cancer and HPV vaccination among adolescent girls aged 10 to 19 years and examine associations between sociodemographic factors and knowledge levels.

Methods

A community-based cross-sectional study was conducted over three months among 180 adolescent girls in the Urban Health Training Centre (UHTC) field practice area of Challapura, Datia, Madhya Pradesh. Participants were selected using systematic random sampling. Data were collected using a semi-structured questionnaire, with descriptive statistics and chi-square tests applied at P < 0.05.

Results

Late adolescents (aged 17 to 19 years) comprised 94 (52.22%) of participants. Only 42 (23.33%) had knowledge of HPV, while 138 (76.67%) were unaware. The internet was the main source of information, reported by 73 (40.48%), followed by friends (43, 23.81%) and health workers (30, 16.67%). Knowledge of cervical cancer (χ² = 39.09, p < 0.001), HPV infection (χ² = 21.00, p < 0.001), and HPV vaccination (χ² = 11.37, p = 0.003) were significantly associated with age. Attitudes indicated moderate perception of disease severity but lower acceptance of vaccination. The main barriers were lack of awareness reported by 126 (70.0%), lack of healthcare recommendations by 57 (31.7%), and high cost by 38 (21.1%).

Conclusion

HPV awareness among adolescent girls was low despite moderately favorable attitudes toward prevention. Targeted educational interventions and increased involvement of healthcare providers are needed to improve vaccine awareness and acceptance in urban primary care settings.

## Introduction

Cervical cancer remains a major global public health concern and is the fourth most common cancer among women worldwide. In 2022, approximately 660,000 new cases and 350,000 deaths were reported, with a disproportionate burden in low- and middle-income countries (LMICs), where access to preventive and early detection services is limited [1]. India contributes substantially to this burden, highlighting persistent challenges in effective prevention, screening, and control strategies.

Persistent infection with high-risk human papillomavirus (HPV), particularly types 16 and 18, is the necessary cause of almost all cervical cancer cases. This strong causal relationship underscores the importance of HPV vaccination as a key primary prevention strategy [2].

Adolescent girls represent a critical target group for HPV vaccination and health education initiatives. However, existing evidence indicates substantial gaps in awareness and knowledge. A community-based study in Western India reported that only 25.2% of adolescent girls were aware of cervical cancer, and only 5.4% had prior knowledge of the HPV vaccine [3]. Similarly, urban-based studies have demonstrated moderate awareness of cervical cancer, while understanding of HPV as a causative factor remains limited and is often associated with higher educational status [4]. Findings from slum populations in Kolkata further highlight poor knowledge, negative attitudes, and low uptake of screening and vaccination services [5].

Interventional studies have shown that structured educational programs can significantly improve awareness and vaccine acceptability among adolescents [6]. However, a persistent gap exists between knowledge and actual preventive practices. Evidence from Nepal suggests that even with adequate awareness, negative attitudes and poor screening behaviors may continue [7]. In Madhya Pradesh, HPV vaccine uptake remains extremely low (3.33%) despite high willingness to receive the vaccine [8]. Encouragingly, studies among urban adolescents have reported substantial improvement in awareness and vaccination intent following targeted educational interventions [9].

Despite the growing body of literature, there is a paucity of data from smaller urban field practice areas such as Urban Health Training Centres (UHTCs), which primarily serve underserved populations and play a key role in delivering primary healthcare services. These settings differ from large urban or institutional populations in terms of access, awareness, and healthcare utilization patterns. Limited evidence is available regarding knowledge, attitudes, and practices (KAP) related to cervical cancer and HPV vaccination among adolescents in such contexts, particularly in central India.

Therefore, the present community-based study was conducted to assess the KAP regarding cervical cancer and HPV vaccination among adolescent girls attending the UHTC in Chhallapura, Datia, Madhya Pradesh. The findings are expected to identify existing gaps and support the development of targeted educational and preventive strategies in urban primary healthcare settings.

## Materials and methods

Study design and setting

An analytical community-based cross-sectional study was conducted in the urban field practice area served by the UHTC, Chhallapura, which functions as an urban outreach unit of the associated medical college. The UHTC provides comprehensive primary healthcare services to a defined urban population and serves as a field practice area for teaching, training, and community-based research activities.

Study duration

The study was conducted over a period of three months, from 1 January 2025 to 31 March 2025, following approval from the Institutional Ethics Committee. The study period included preparatory activities, pilot testing of the study tool, participant recruitment, data collection, data verification, and statistical analysis.

Study population

The study population comprised adolescent girls aged 10 to 19 years residing in the UHTC Chhallapura field practice area. Permanent residents, defined as individuals who had been living in the study area for at least six months prior to the survey, were considered eligible for participation to ensure adequate representation of the target population.

Inclusion criteria

Adolescent girls aged 10 to 19 years who were permanent residents of the study area and willing to participate were included in the study. Written informed consent was obtained from participants aged 18 to 19 years. For participants younger than 18 years, written assent was obtained along with consent from a parent or legal guardian.

Exclusion criteria

Adolescent girls who were critically ill at the time of the survey, had cognitive or mental impairments that limited their ability to respond appropriately to the questionnaire, or declined participation were excluded from the study.

Sample size determination

The sample size was calculated using the single population proportion formula:

$N=\frac{Z^{2}p(1-p)}{d^{2}}$

Where, N = required sample size, Z = standard normal deviate corresponding to the desired confidence level (1.96 for 95% confidence level), p = expected prevalence of adequate knowledge regarding cervical cancer and HPV vaccination (20.83%) based on previous literature [8] and d = absolute precision (6%). Substituting these values yielded a minimum sample size of 175.97, which was rounded off to 180 participants.

As the primary objective of the study was to estimate the level of kKAP regarding cervical cancer and HPV vaccination among adolescent girls, a prevalence-based sample size estimation was considered appropriate.

Sampling technique and procedure

A systematic random sampling technique was employed for participant selection. A complete and updated household list obtained from UHTC records served as the sampling frame. The sampling interval (k) was calculated by dividing the total number of households in the study area by the required sample size.

The first household was selected randomly using a random number table, after which every kth household was visited. During each household visit, eligibility screening was performed. If an eligible adolescent girl was identified, she was invited to participate in the study after obtaining appropriate consent.

In households with more than one eligible adolescent girl, one participant was selected using the lottery method to minimize clustering effects. In cases where no eligible participant was available or consent was not obtained, the next immediate household was approached. This process continued until the desired sample size was achieved.

Data collection tool and procedure

Data were collected using a predesigned, semi-structured, interviewer-administered questionnaire consisting predominantly of closed-ended questions (Appendix). The questionnaire was developed following an extensive review of relevant literature and previously published instruments assessing KAP related to cervical cancer and HPV vaccination.

Prior to the main study, the questionnaire was pretested among 36 adolescent girls from a population similar to, but outside, the study area. The pretesting exercise was conducted to assess clarity, comprehensibility, feasibility, and appropriateness of the questions. Based on the feedback obtained during pretesting, necessary modifications were made before finalization of the questionnaire.

The final questionnaire comprised sections covering sociodemographic characteristics, knowledge regarding cervical cancer and HPV vaccination, attitudes towards cervical cancer prevention and HPV vaccination and practices related to cervical cancer awareness and HPV vaccination.

Data were collected through face-to-face interviews conducted by trained investigators using standardized interviewing techniques to minimize interviewer bias and ensure uniformity in data collection. Privacy was maintained throughout the interview process, and confidentiality of participant information was strictly ensured.

Handling of missing data

Completed questionnaires were reviewed daily for completeness and consistency by the principal investigator. Questionnaires containing more than 10% missing responses were excluded from analysis. For questionnaires with minimal missing data, pairwise deletion was employed during statistical analysis. No imputation techniques were used.

Data management and statistical analysis

Collected data were coded and entered into Microsoft Excel 2016 (Redmond, WA, USA) using a predefined coding scheme. Double data entry and random verification of records were performed to minimize entry errors and ensure data quality. Data analysis was carried out using Jamovi software version 2.6.44 (The Jamovi Project, Sydney, Australia). Quantitative variables were summarized using mean and standard deviation, whereas categorical variables were presented as frequencies and percentages. Associations between categorical variables were assessed using the Chi-square test or Fisher's exact test, as appropriate. All statistical tests were two-tailed, and a p-value of less than 0.05 was considered statistically significant.

Ethical considerations

Ethical approval for the study was obtained from the Institutional Ethics Committee of Biomedical and Health Research in Human Participants (IECBMHR) (Approval No. 102/CM/GMC/IECBHMR/2024/Version-1) prior to commencement of the study. Written informed consent was obtained from all participants aged 18 to 19 years. For participants younger than 18 years, written assent was obtained in addition to consent from a parent or legal guardian. Participation was entirely voluntary, and participants were informed of their right to withdraw from the study at any stage without any consequences. Confidentiality and anonymity of participant information were maintained throughout the study. Data were stored securely and used solely for research purposes.

## Results

A total of 180 adolescent girls were included in the analysis. More than half of the participants belonged to the late adolescent age group (52.22%), followed by mid-adolescents (31.67%) and early adolescents (16.11%). Regarding educational status, 35.56% of participants were illiterate, while 17.78% had completed primary education, 16.11% secondary education, 12.78% higher secondary education, and 17.78% were graduates or above. A family history of cervical cancer was reported by 1.11% of the participants. Overall, only 23.33% of participants reported prior knowledge of HPV, whereas 76.67% were unaware. Among the reported sources of knowledge, the internet was the most common source (40.48%), followed by friends (23.81%), health workers (16.67%), television (11.90%), and newspapers (7.14%) (Table 1).

**Table 1:** Sociodemographic Characteristics and Awareness of Human Papillomavirus (HPV) Among Adolescent Girls (N = 180) The table presents the distribution of study participants according to age, educational status, family history of cervical cancer, source of knowledge regarding HPV, and knowledge regarding HPV. Data are expressed as number of participants with corresponding percentages in parentheses [N (%)].

Variable	Category	N (%)
Source of Knowledge of HPV	Newspaper	13 (7.14)
	Television	21 (11.90)
	Health worker	30 (16.67)
	Friends	43 (23.81)
	Internet	73 (40.48)
Age group (in years)	10-13	29 (16.11)
	14-16	57 (31.67)
	17-19	94 (52.22)
Educational Status of Participants	Illiterate	64 (35.56)
	Primary	32 (17.78)
	Secondary	29 (16.11)
	Higher secondary	23 (12.78)
	Graduate or above	32 (17.78)
Family History of Cervical Cancer	Yes	2 (1.11)
	No	178 (98.89)
Knowledge Regarding HPV	Yes	42 (23.33)
	No	138 (76.67)

Knowledge of cervical cancer was significantly associated with age group (χ² = 39.09, p < 0.001). Similarly, knowledge of HPV infection varied significantly across age groups (χ² = 21.0, p < 0.001). Knowledge of HPV vaccination also showed a significant association with age group (χ² = 11.37, p = 0.003) (Table 2).

**Table 2:** Association Between Age Group and Knowledge of Cervical Cancer, Human Papillomavirus (HPV) Infection, and HPV Vaccination Among Adolescent Girls (N = 180) Values are expressed as number (percentage) within each age group. Percentages were calculated column-wise using the total number of participants in each age category as the denominator (early adolescents n = 29, mid adolescents n = 57, late adolescents n = 94). The chi-square (χ²) test was applied to assess the association between age group and knowledge variables. A p-value of less than 0.05 was considered statistically significant. Statistical significance is indicated as *p < 0.05, **p < 0.01, and ***p < 0.001.

Parameter	Response	Early adolescent (10–13 years) n = 29	Mid adolescent (14–16 years) n = 57	Late adolescent (17–19 years) n = 94	χ²	p-value
Knowledge of cervical cancer	Yes	5 (17.2%)	9 (15.8%)	39 (41.5%)	39.09	<0.001***
	No	24 (82.8%)	48 (84.2%)	55 (58.5%)		
Knowledge of HPV infection	Yes	3 (10.3%)	12 (21.1%)	27 (28.7%)	21	<0.001***
	No	26 (89.7%)	45 (78.9%)	67 (71.3%)		
Knowledge regarding HPV vaccine	Yes	8 (27.6%)	6 (10.5%)	21 (22.3%)	11.37	0.003**
	No	21 (72.4%)	51 (89.5%)	73 (77.7%)		

The perception of cervical cancer as a serious health issue had a mean score of 1.32 ± 1.17. The perceived effectiveness of the HPV vaccine had a mean score of 1.60 ± 1.23, while acceptance of universal HPV vaccination for girls had a lower mean score of 1.09 ± 1.20. The mean score for perceived safety of the HPV vaccine was 1.34 ± 1.18. Cost as a barrier to vaccination showed a mean score of 1.33 ± 1.15, whereas support for school-based HPV vaccination had the lowest mean score (1.05 ± 1.10). The mean scores for views on gender-specific HPV vaccination and the role of the government in raising vaccine awareness were 1.47 ± 1.23 and 1.36 ± 1.06, respectively (Table 3).

**Table 3:** Attitude Assessment Regarding Cervical Cancer and Human Papillomavirus (HPV) Vaccination Among Adolescent Girls (N = 180) Values are presented as number (percentage) of participants for each response category. Percentages were calculated using the total sample size (N = 180) as the denominator. Responses were recorded on a five-point Likert scale ranging from 0 to 4, where higher scores indicate more favorable attitudes toward cervical cancer prevention and HPV vaccination. Mean scores with standard deviations (Mean ± SD) were calculated for each item to summarize overall participant attitudes.

Item No	Details	Score 0	Score 1	Score 2	Score 3	Score 4	Mean±SD
1	Is cervical cancer a serious issue?	56 (31.11)	49 (27.22)	47 (26.11)	18 (10)	10 (5.56)	1.32 ± 1.17
2	Is the HPV vaccine effective?	43 (23.89)	42 (23.33)	53 (29.44)	28 (15.56)	14 (7.78)	1.60 ± 1.23
3	Should all girls get the HPV vaccine?	74 (41.11)	54 (30)	21 (11.67)	23 (12.78)	8 (4.44)	1.09 ± 1.2
4	Is the HPV vaccine safe?	59 (32.78)	37 (20.56)	56 (31.11)	19 (10.55)	9 (5)	1.34 ± 1.18
5	Does cost hinder vaccination?	57 (31.67)	40 (22.22)	57 (31.67)	18 (10)	8 (4.44)	1.33 ± 1.15
6	Should HPV vaccination be school-based?	74 (41.11)	47 (26.11)	41 (22.78)	12 (6.67)	6 (3.33)	1.05 ± 1.1
7	Should only women get the HPV vaccine?	51 (28.33)	43 (23.88)	47 (26.11)	28 (15.56)	11 (6.11)	1.47 ± 1.23
8	Should the government raise vaccine awareness?	48 (26.67)	49 (27.22)	55 (30.56)	26 (14.44)	2 (1.11)	1.36 ± 1.06

**Figure 1:**
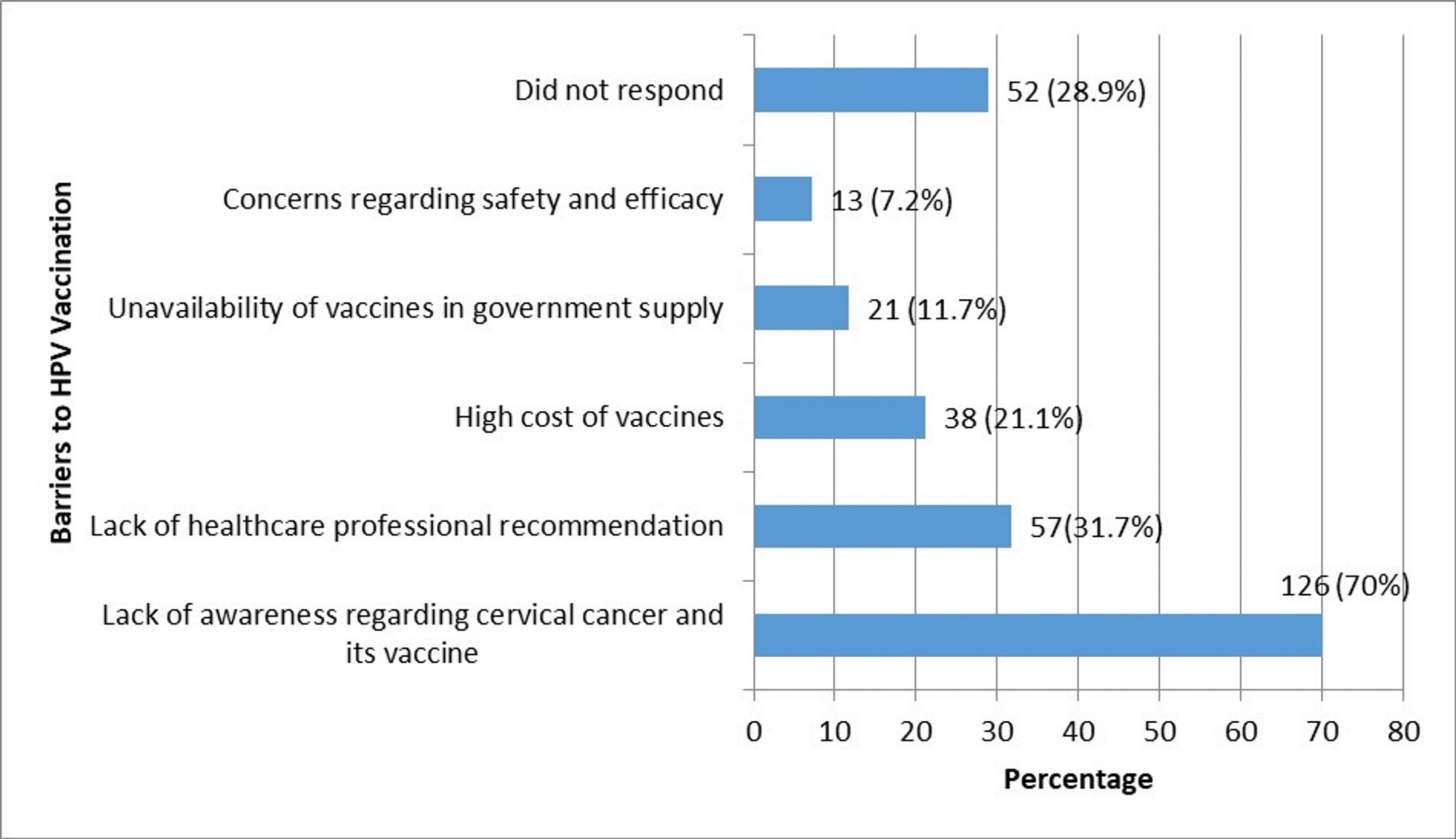
Barriers to Human Papillomavirus (HPV) Vaccination Among Adolescent Girls (N = 180) Values are presented as number and percentage [N (%)] reporting each barrier to HPV vaccination (N = 180). Multiple responses were allowed; therefore, percentages may not sum to 100%. Lack of awareness regarding cervical cancer and its vaccine was the most commonly reported barrier, followed by lack of healthcare professional recommendation and high cost of vaccines.

No participant in the study reported having received the HPV vaccine; therefore, no HPV vaccination practices were observed in the study sample. Lack of awareness regarding cervical cancer and its vaccine was the most commonly reported barrier (70.0%), followed by lack of healthcare professional recommendation (31.7%). High vaccine cost was reported by 21.1% of participants, while unavailability of vaccines in the government supply was reported by 11.7%. Concerns related to vaccine safety and efficacy were reported by 7.2% of participants, and 28.9% did not respond to the question (Figure 1).

## Discussion

The present study demonstrated low awareness of HPV and its vaccine among adolescent girls attending a UHTC in Datia, Madhya Pradesh, with only 23.33% reporting prior knowledge of HPV. This level of awareness was substantially lower than that reported in several international and national studies. A systematic review and meta-analysis among Ethiopian schoolgirls reported a pooled prevalence of good knowledge of HPV vaccination of 55.12%, which is more than double the proportion observed in the present study [10]. Similarly, community-based data from Ethiopia showed that 46.4% of women had knowledge about cervical cancer, indicating comparatively better awareness even among adult populations in low-resource settings [11]. These findings suggest that adolescent girls in the present setting may have limited exposure to structured health information, particularly regarding reproductive health.

Age was significantly associated with knowledge of cervical cancer, HPV infection, and HPV vaccination in the present study, with late adolescents demonstrating better awareness than early adolescents. This pattern is consistent with findings from Indonesia, where younger age groups exhibited poorer knowledge but more favorable attitudes, indicating a gradual accumulation of information with increasing age and education [12]. A similar association has been reported among nursing students in India, where senior students had higher odds of adequate knowledge compared to first-year students [13]. These observations highlight the importance of introducing cervical cancer and HPV-related education at an earlier stage of adolescence.

The Internet was the most common source of information (40.48%) in the present study, whereas health workers accounted for only 16.67% of responses. This reliance on informal and digital sources is comparable to the findings from Indonesian urban communities, where knowledge remains fragmented despite widespread media exposure [12]. In contrast, studies from Nigeria and Ethiopia have demonstrated that community sensitization and school-based health education programs led by trained personnel significantly improve awareness and vaccine acceptance [10,14]. The limited role of healthcare providers observed in the present study may contribute to persistent misconceptions and reduced confidence in HPV vaccination.

The findings indicate that while people perceive cervical cancer as somewhat serious and believe in the efficacy of the vaccine, they are less inclined to accept vaccines administered universally or in schools. Similar trends are observed in Ethiopia, where, despite moderate knowledge, only 45.34% held a positive attitude and 42.05% received the vaccine [10]. In India, Shah et al. discovered that although people were aware of HPV, awareness of the vaccine itself increased their likelihood of wanting it, underscoring the importance of clear information [15]. The low scores for vaccine safety and school vaccinations in this study suggest uncertainty rather than opposition.

None of the participants in this study reported receiving the HPV vaccine, indicating a complete absence of vaccination practices in the study population. This finding highlights the combined impact of low awareness, limited healthcare provider engagement, and systemic barriers in preventing the adoption of preventive measures.

Lack of awareness (70.0%) was the most frequently reported barrier to HPV vaccination, followed by a lack of healthcare professional recommendations (31.7%). Similar barriers have been identified in Zimbabwe, where inadequate knowledge and limited access to services restricted vaccine uptake despite high acceptability [16]. Cost-related barriers (21.1%) and lack of government supply (11.7%) observed in the present study are consistent with evidence from India, where out-of-pocket expenditure and unclear national guidelines limit routine HPV vaccination recommendations by health care providers [17]. Studies from rural Mysore and Uttar Pradesh further demonstrate that free vaccine provision and strong family support significantly enhance vaccine acceptance and uptake [18,19].

Overall, compared to global and national evidence, the findings of the present study reflect a more pronounced knowledge deficit and a cautious attitude toward HPV vaccination among adolescent girls. In contrast to settings where structured school-based education and organized health interventions are implemented, the present findings underscore the need for early, provider-led education, community engagement, and improved accessibility of vaccination services. Strengthening these components is essential to bridge the gap between awareness, attitudes, and preventive practices in similar urban health settings in the future.

Limitations

The present study has several limitations. First, the cross-sectional design limits the ability to establish causal relationships between sociodemographic factors, knowledge, attitudes, and vaccination practices. Second, the study was conducted in the field practice area of a single Urban Health Training Centre, which may limit the generalizability of the findings to adolescent populations with different sociodemographic and healthcare characteristics. Third, although the questionnaire was pretested among 36 participants from a similar population outside the study area and necessary modifications were made, formal psychometric evaluation, including validity and reliability testing, was not performed. Therefore, measurement error and misclassification cannot be completely excluded. Fourth, a substantial proportion of participants were illiterate, and the data were collected through self-reported responses during interviews, which may have introduced information bias due to misunderstanding of questions, recall bias, or social desirability bias. Fifth, vaccination status was not verified using immunization records and relied solely on participant responses, which may not accurately reflect the true vaccination status of participants. Finally, parental knowledge, attitudes, and decision-making processes, which are known to influence adolescent health-seeking behavior and HPV vaccine acceptance, were not assessed in the present study. Future studies incorporating parental perspectives and employing validated assessment tools across multiple settings would provide a more comprehensive understanding of factors influencing HPV vaccination uptake among adolescents.

## Conclusions

The present study indicates a low level of awareness regarding cervical cancer, HPV infection, and its vaccination among adolescent girls, with particularly poor knowledge among early adolescents. Although awareness improved with age, significant gaps and misconceptions persisted across all groups. Digital media emerged as the primary source of information, while the limited role of healthcare professionals reflects missed opportunities for accurate counselling. These findings highlight the need for age-appropriate, targeted educational interventions that integrate digital platforms with active healthcare provider involvement. Strengthening structured health education at community and school levels is essential to improve awareness, address misconceptions, and promote informed decision-making regarding HPV prevention and vaccination.

## Appendices

**Table 4:** Questionnaire for Assessing Knowledge, Attitudes, and Practices Related to Cervical Cancer and Human Papillomavirus (HPV) Vaccination Among Adolescent Girls This semi-structured questionnaire was designed to assess sociodemographic characteristics and the knowledge, attitudes, and practices related to cervical cancer, human papillomavirus infection, and HPV vaccination among adolescent girls. The tool is divided into six sections: sociodemographic profile (Items 1–7), awareness and knowledge (Items 8–21), knowledge of cervical cancer (Items 22–23), knowledge of HPV vaccination (Items 24–32), attitude toward HPV vaccination (Items 33–42), and vaccination-related practices (Items 43–52). Responses include dichotomous (yes/no), multiple-choice, and Likert scale formats. Knowledge-related items assess awareness of disease, transmission, prevention, and vaccination, while attitude items use a graded response scale ranging from strongly agree to strongly disagree or similar ordinal options. Practice-related items capture actual behaviors, healthcare consultation, and barriers to vaccination. This instrument allows comprehensive evaluation of gaps in awareness, perception, and preventive practices related to HPV and cervical cancer.

Section A: Sociodemographic Profile				
Q. No.			Question	Response Options
1			Name / Daughter of	__________
2			Age group (in years)	10–13 / 14–16 / 17–19
3			Residence	Rural / Urban
4			Religion	Hindu / Muslim / Other
5			Caste	General / OBC / SC / ST
6			Education level	Illiterate / Up to 8th grade / 10th grade / 12th grade / Graduation and above
7			Socioeconomic status (According to Modified Kuppuswamy scale)	Upper / Upper middle / Middle / Lower middle / Lower
Section B: Awareness and Knowledge				
8			Have you ever heard about HPV infection?	Yes / No
9			If yes, what was your source of information?	Newspaper / Radio / Television / Internet / Books / Health worker / Friends
10			Do you know about the HPV vaccine?	Yes / No
11			Have you ever heard of cervical cancer?	Yes / No
12			Do you think cervical cancer is common in India?	Yes / No / Don’t know
13			Do you think HPV infection causes cervical cancer?	Yes / No / Don’t know
14			Do you think HPV spreads through sexual contact?	Yes / No / Don’t know
15			Can HPV infection be treated with medicines?	Yes / No / Don’t know
16			Do you think HPV infection is self-limiting?	Yes / No / Don’t know
17			Can HPV be detected through medical tests?	Yes / No / Don’t know
18			Can HPV infection be prevented using barrier methods (e.g., condoms)?	Yes / No / Don’t know
19			Should women undergo screening for HPV infection?	Yes / No / Don’t know
20			Is HPV infection common in the general population?	Yes / No / Don’t know
21			Does HPV infection cause symptoms in women?	Yes / No / Don’t know
Section C: Knowledge of Cervical Cancer				
22			Symptoms of cervical cancer	Post-coital bleeding / Intermenstrual or postmenopausal bleeding / Foul-smelling vaginal discharge / Pelvic pain or pain during intercourse / Don’t know
23			Risk factors of cervical cancer	HIV infection / Long-term use of oral contraceptives / Multiple sexual partners / Don’t know
Section D: Knowledge of HPV Vaccination				
24	Are you aware of the cervical cancer vaccine?			Yes / No / Don’t know
25	Do you know the names of HPV vaccines?			Yes / No (If yes, specify: ________)
26	At what age should HPV vaccination be taken?			9–14 years / 15–26 years / Don’t know
27	How many doses are required?			1 / 2 / 3 / Don’t know
28	Can the vaccine be given to both males and females?			Yes / No / Don’t know
29	Does the HPV vaccine completely prevent cervical cancer?			Yes / No / Partially
30	Have you received information from a doctor or health worker?			Yes / No
31	Has anyone in your family or friends taken the HPV vaccine?			Yes / No / Don’t know
32	How aware are you about HPV vaccination?			Scale 1–5 (1 = Not aware, 5 = Fully aware)
Section E: Attitude Towards HPV Vaccination				
Q. No.		Question		Response Options
33		Do you think cervical cancer is a serious health problem?		Yes / No / Don’t know
34		HPV vaccine is effective in preventing cervical cancer		Strongly agree / Agree / Disagree / Strongly disagree
35		All girls should receive HPV vaccination		Yes / No / Don’t know
36		HPV vaccine is safe		Strongly agree / Agree / Disagree / Strongly disagree
37		Cost prevents people from taking the vaccine		Yes / No / Don’t know
38		HPV vaccine should be included in school programs		Strongly agree / Agree / Disagree / Strongly disagree
39		Only females should receive HPV vaccine		Yes / No / Don’t know
40		Government should increase awareness about HPV vaccination		Strongly agree / Agree / Disagree / Strongly disagree
41		Cultural or religious beliefs affect vaccine acceptance		Yes / No / Don’t know
42		Would you take or recommend HPV vaccination?		Yes / No / Will consider
Section F: Practices Related to HPV Vaccination				
Q. No.		Question		Response Options
43		Have you or your family member received HPV vaccination?		Yes / No / Don’t know
44		If yes, when was it taken?		Last 6 months / Last 1 year / More than 1 year / Don’t remember
45		Did you consult a doctor before vaccination?		Yes / No
46		Where was the vaccination received?		Government facility / Private facility / Health camp / Other
47		Have girls aged 9–14 years in your family received the vaccine?		Yes / No / Don’t know
48		If not vaccinated, what was the reason?		Lack of awareness / High cost / Unavailability / Safety concerns / Other
49		Have you heard about HPV vaccination through any awareness program?		Yes / No
50		Do you undergo regular health check-ups including screening?		Yes / No
51		Source of vaccine information		Doctor/health worker / TV/Radio / Social media / Friends/family / Other
52		Do you know about the next dose schedule?		Yes / No / Not applicable

## References

[1] (2026). Cervical Cancer: Fact Sheet. https://www.who.int/news-room/fact-sheets/detail/cervical-cancer.

[2] (2026). Cervical cancer—causes, risk factors, and prevention. https://www.cancer.gov/types/cervical/causes-risk-prevention.

[3] Vachhani S, Bhatt R (2023). An interventional study to assess knowledge regarding cervical cancer and associated preventive measures among adolescent girls of an urban area in Western India. Proc Int Conf Public Health.

[4] Ahlawat P, Batra N, Sharma P, Kumar S, Kumar A (2018). Knowledge and attitude of adolescent girls and their mothers regarding cervical cancer: a community-based cross-sectional study. J Midlife Health.

[5] Das A, Sarkar S, Nawal H (2025). Knowledge, attitude and practice on cervical cancer screening and human papillomavirus vaccination among adolescent girls residing in a slum of Kolkata. Bangabandhu Sheikh Mujib Med Univ J.

[6] Sneha LM, Scott JX, Kennedy AS (2018). Impact of a brief school-based educational intervention to increase the knowledge about HPV vaccination among adolescent girls. Int J Healthc Educ Med Inform.

[7] Chaudhary K, Rai G, Karn BK (2022). Health literacy on human papillomavirus, its vaccination and risk factors of cervical cancer among adolescent girls. South Asian Res J Nurs Health Care.

[8] Jatav P, Sirohi S, Dixit S (2024). A cross-sectional study to assess knowledge, attitude and practice towards cervical cancer, human papilloma virus (HPV) infection and HPV vaccine among 9th to 12th grade school girls in Indore. Indian J Public Health Res Dev.

[9] Ramavath KK, Olyai R (2013). Knowledge and awareness of HPV infection and vaccination among urban adolescents in India: a cross-sectional study. J Obstet Gynaecol India.

[10] Addisu D, Gebeyehu NA, Belachew YY (2023). Knowledge, attitude, and uptake of human papillomavirus vaccine among adolescent schoolgirls in Ethiopia: a systematic review and meta-analysis. BMC Womens Health.

[11] Tsegay A, Araya T, Amare K, G/Tsadik F (2020). Knowledge, attitude, and practice on cervical cancer screening and associated factors among women aged 15-49 years in Adigrat town, northern Ethiopia, 2019: a community-based cross-sectional study. Int J Womens Health.

[12] Winarto H, Habiburrahman M, Dorothea M (2022). Knowledge, attitudes, and practices among Indonesian urban communities regarding HPV infection, cervical cancer, and HPV vaccination. PLoS One.

[13] Chauhan S, Tiwari SK, Dubey V, Tripathi P, Pandey P, Singh A, Choudhary NP (2025). Knowledge, attitude, and reasons for non-uptake of human papilloma virus vaccination among nursing students. BMC Med.

[14] Egbon M, Ojo T, Aliyu A, Bagudu ZS (2022). Challenges and lessons from a school-based human papillomavirus (HPV) vaccination program for adolescent girls in a rural Nigerian community. BMC Public Health.

[15] Shah PM, Ngamasana E, Shetty V (2022). Knowledge, attitudes and HPV vaccine intention among women in India. J Community Health.

[16] Zibako P, Tsikai N, Manyame S, Ginindza TG (2021). Knowledge, attitude and practice towards cervical cancer prevention among mothers of girls aged between 9 and 14 years: a cross sectional survey in Zimbabwe. BMC Womens Health.

[17] Kataria I, Siddiqui M, Treiman K (2022). Awareness, perceptions, and choices of physicians pertaining to human papillomavirus (HPV) vaccination in India: a formative research study. Vaccine X.

[18] Coursey K, Muralidhar K, Srinivas V (2024). Acceptability of HPV vaccination for cervical cancer prevention amongst emerging adult women in rural Mysore, India: a mixed-methods study. BMC Public Health.

[19] Holroyd TA, Yan SD, Srivastava V (2022). Designing a pro-equity HPV vaccine delivery program for girls who have dropped out of school: community perspectives from Uttar Pradesh, India. Health Promot Pract.

